# Human olfactory receptor responses to odorants

**DOI:** 10.1038/sdata.2015.2

**Published:** 2015-02-03

**Authors:** Joel D Mainland, Yun R Li, Ting Zhou, Wen Ling L Liu, Hiroaki Matsunami

**Affiliations:** 1 Monell Chemical Senses Center, 3500 Market Street, Philadelphia, Pennsylvania 19104, USA; 2 Department of Molecular Genetics and Microbiology, Duke University Medical Center, Research Drive, Durham, North Carolina 27710, USA; 3 Department of Neuroscience, University of Pennsylvania School of Medicine, Philadelphia, Pennsylvania 19104, USA; 4 Department of Neurobiology and Duke Institute for Brain Sciences, Duke University Medical Center, Research Drive, Durham, North Carolina 27710, USA

**Keywords:** Behavioural genetics, Olfactory receptors

## Abstract

Although the human olfactory system is capable of discriminating a vast number of odors, we do not currently understand what chemical features are encoded by olfactory receptors. In large part this is due to a paucity of data in a search space covering the interactions of hundreds of receptors with billions of odorous molecules. Of the approximately 400 intact human odorant receptors, only 10% have a published ligand. Here we used a heterologous luciferase assay to screen 73 odorants against a clone library of 511 human olfactory receptors. This dataset will allow other researchers to interrogate the combinatorial nature of olfactory coding.

## Background & Summary

Previous functional analysis of olfactory receptors (ORs) in olfactory neurons and in heterologous cells found that different odorants are recognized by unique, but overlapping ensembles of ORs^1–4^. These findings suggest that specific patterns of ORs activated by an odorant code for the odorant’s identity, but there are few, if any, explicit predictions relating OR activity patterns to olfactory perception.

Matching mammalian ORs to ligands has seen limited success, and the picture is even worse when considering human ORs; ligands have been published for only 49 of the approximately 400 intact human ORs^5–21^. This lack of data is a critical bottleneck in the field; matching ligands to ORs is critical for understanding the olfactory system at all levels and is essential for building viable models of olfaction. The characterization of OR responses to ligands in the empty neuron system of *Drosophila melanogaster* ^22^ has allowed researchers in the field to choose rationally diverse odorant sets^23^ and specifically manipulate subpopulations of ORs to dissect olfactory coding^24,25^. Extending this idea by matching odorants to human ORs has the added advantage that humans can directly communicate their perception of odor intensity, pleasantness, and quality.

In addition, understanding the role of a single OR in olfactory perception allows us to look at evolutionary changes in OR genes in a new light. For example, the knowledge that Tas1r2 is a pseudogene in seven of twelve species in the order *Carnivora* ^26^ is difficult to interpret in isolation. The knowledge that Tas1r2 is the primary mediator of sweet taste in mice, however, suggests that carnivores do not need to taste sweet and therefore there is no selective pressure on the gene. As several genome sequencing projects are examining both genetic variation within humans^27^ and across species^28^, understanding the role of OR genes in olfactory perception becomes crucial to the interpretation of how and why genetic changes occur over the course of evolution.

In a recent manuscript we conducted a high-throughput screen of 511 human odorant receptors against 73 odorants^15^. The resulting screen identified agonists for 27 odorant receptors, including 18 that were previously orphan receptors. We went on to characterize how genetic variation in these receptors alters both *in vitro* responses and influences olfactory perception. In this manuscript we present the full screening data to permit wider reuse and reanalysis.

In summary, this dataset addresses a major bottleneck in the field, namely how the physical stimulus in olfaction is transduced into receptor responses. In addition, the G-protein coupled receptor class accounts for approximately 50% of therapeutic drug targets^29^. The ORs, being GPCRs, offer the opportunity to examine the strategies employed by this receptor class to recognize a wide variety of ligand structural features and thus will provide insight into fundamental principles of ligand recognition by GPCRs. Matching odorants to ORs will provide a valuable resource to the field and allow more specific explorations of links between odor, behavior and ecology.

## Methods

These methods are expanded from descriptions in our previous work^15^.

### Cloning

OR open reading frames were amplified from genomic DNA using Phusion polymerase and subcloned into *pCI* expression vectors (Promega) containing the first 20 residues of human rhodopsin (Rho tag). Human ORs were amplified from the pooled genomic DNA of 20 participants from the International Hapmap Consortium, while mouse ORs were amplified from the genomic DNA of C57/BL6 mice. The sequences of the cloned receptors were verified by sequencing (3100 Genetic Analyzer, Applied Biosystems). Clones that were present in the 1000 Genomes Project, but not cloned from our pooled genomic DNA sample, were created using an overlap extension polymerase chain reaction protocol^30^.

### Luciferase assay

The Dual-Glo Luciferase Assay System (Promega) was used to measure receptor responses as previously described^31^. Hana3A cells were transfected with 5 ng/well of RTP1S^32^, 5 ng/well of pRL-SV40, 10 ng/well of CRE-luciferase, 2.5 ng/well of M3 (ref. 33), and 5 ng/well of odorant receptor. 1 M odorant stocks are diluted in DMSO. 24 hours following transfection, transfection media was removed and replaced with the appropriate concentration of odor diluted from the 1 M stocks in CD293 (Gibco). Four hours following odor stimulation luminescence was measured using a Polarstar Optima plate reader (BMG). All luminescence values were divided by the Renilla Luciferase activity to control for transfection efficiency in a given well. Data were analyzed with Microsoft Excel, GraphPad Prism 4, and MATLAB.

### Primary screen design

**Figure 1:**
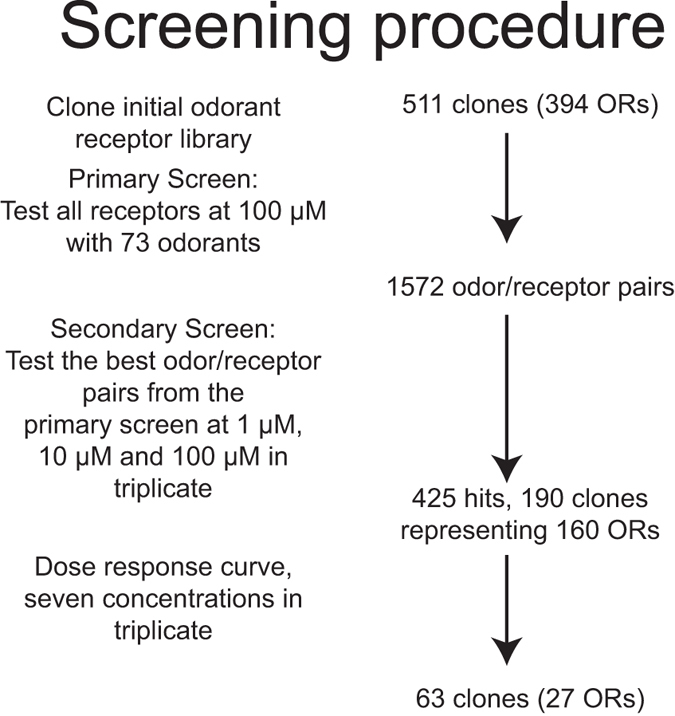
Outline of the screening procedure. This figure was reprinted from our previous publication^15^, where it was included as Supplementary Figure 1.

Our screen design is outlined in Figure 1. In the primary screen we stimulated 511 human ORs with 73 odorants used in previous psychophysical testing^12,34^. We applied the majority of odorants at a concentration of 100 μM. All plates in the primary screen included 85 test wells, five broadly-tuned odorant receptors (Olfr1079, OR2W1, Olfr1377, Olfr73, Olfr1341), and six wells transfected with Oflr544 which served as a standard. Of the six wells transfected with Olfr544, three were challenged with the diluent (CD293) and three were challenged with 10 μM of a known ligand for Olfr544 (nonanedioic acid). Each screening run consisted of twelve plates where each of the 96-wells were transfected with the same set of receptors. One plate had no odor in all test wells and served as a baseline. The other eleven plates were each challenged with a different test odor.

### Secondary screen design

To rank hits from the Primary Screen we standardized each plate, setting the mean Olfr544 response to nonanedioic acid minus the mean Olfr544 response to the no-odor control to a value of 1. We then subtracted the baseline response for each receptor from the no-odor plate from the response to the odor challenge and ranked the resulting values. We selected the top 5% of odorant/receptor pairs from the primary screen, although not more than the top ten ligands for a given receptor. We then performed a secondary screen in which each odorant receptor was tested against a no-odor control as well as 1, 10 and 100 μM of odor. Each comparison was performed in triplicate, where each measure was collected from separate wells, but each well contained cells from the same parent plate of cells. Note that we began the secondary screen before completion of the entire primary screen, so some odor/receptor combinations outside of the overall top 5% were tested.

### Dose-response design

**Figure 2:**
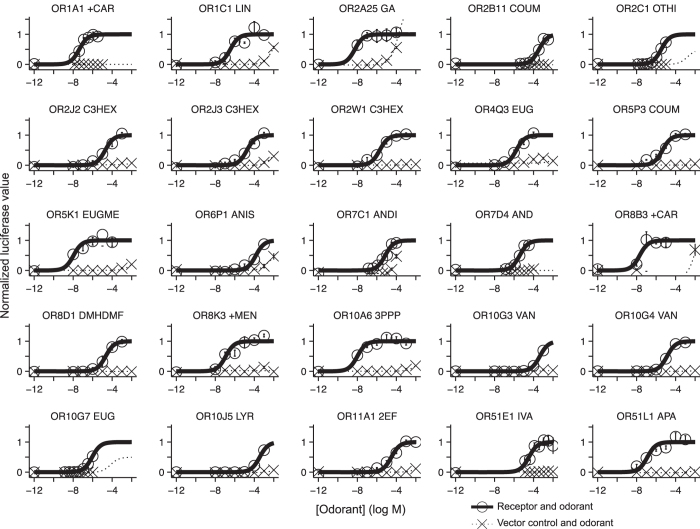
Normalized dose-response curves of the receptor encoded by the most common functional allele for 25 receptors. The responses of cells transfected with either a plasmid encoding the indicated odorant receptor or an empty vector to the indicated odorants. Responses have been normalized such that each receptor has a minimum response of zero and a maximum response of one. Error bars, s.e.m. over three replicates. Abbreviations for the odorants are as follows: +CAR are shown, (+)-carvone; LIN, linalool; GA, geranyl acetate; COUM, coumarin; OTHI, octanethiol; C3HEX, cis-3-hexen-1-ol; EUG, eugenol; EUGME, eugenol methyl ether; ANIS, anisaldehyde; ANDI, 4,16-androstadien-3-one; AND, 5α-androst-16-en-3-one; DMHDMF, caramel furanone; +MEN, (+)-menthol; 3PPP, 3-phenyl propyl propionate; VAN, vanillin; LYR, lyral; 2EF, 2-ethyl fenchol; IVA, isovaleric acid; APA, allyl phenyl acetate. This figure was modified from our previous publication^15^, where it was included as Figure 1.

**Figure 3:**
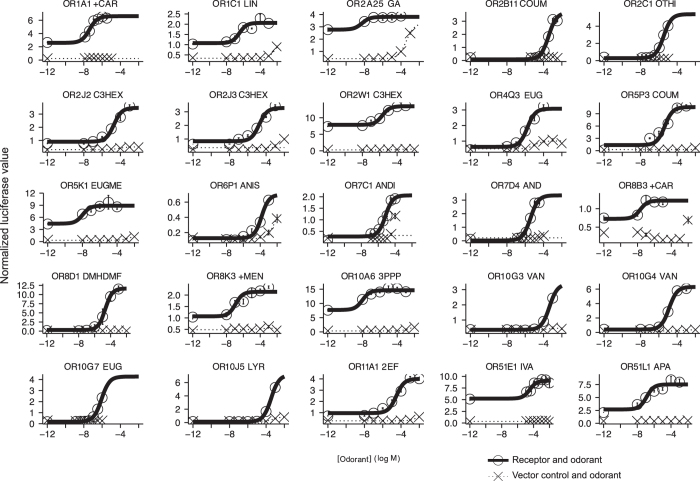
Dose-response curves of the receptor encoded by the most common functional allele for 25 receptors. The responses of cells transfected with either a plasmid encoding the indicated odorant receptor or an empty vector to the indicated odorants. Error bars, s.e.m. over three replicates. Abbreviations for the odorants are as follows: +CAR are shown, (+)-carvone; LIN, linalool; GA, geranyl acetate; COUM, coumarin; OTHI, octanethiol; C3HEX, cis-3-hexen-1-ol; EUG, eugenol; EUGME, eugenol methyl ether; ANIS, anisaldehyde; ANDI, 4,16-androstadien-3-one; AND, 5α-androst-16-en-3-one; DMHDMF, caramel furanone; +MEN, (+)-menthol; 3PPP, 3-phenyl propyl propionate; VAN, vanillin; LYR, lyral; 2EF, 2-ethyl fenchol; IVA, isovaleric acid; APA, allyl phenyl acetate. This figure was modified from our previous publication^15^, where it was included as Figure 1.

We then constructed dose-response curves using concentrations ranging from 10 nM to 10 mM for the odor/receptor pairs that were significantly different from baseline in the Secondary Screen. Each odorant receptor-odorant dose was tested in triplicate, where each measure was collected from separate wells, but each well contains cells from the same parent plate of cells. A vector-only control was included for each odorant. We fit the data to a sigmoidal curve. We counted an odorant as an agonist if the 95% confidence intervals of the top and bottom parameters did not overlap, the standard deviation of the fitted log EC50 was less than 1 log unit, and the extra sums-of-squares test confirmed that the odorant activated the receptor significantly more than the control, which was transfected with an empty vector. This data identified 25 odorant receptors with a significant response to at least one agonist^15^ (Figures 2 and 3).

## Data Records

The data for this manuscript have been deposited in figshare (Data Citation 1). A summary of the clones tested in each phase of the screen is presented in Supplementary Table 1.

### Data record 1—primary screen

The raw screening results are presented in a tab-separated values file (Data Citation 1). Each row represents an experiment from a single well.

***Plate***. A unique ID for a 96-well plate on a given date.

**Figure 4:**
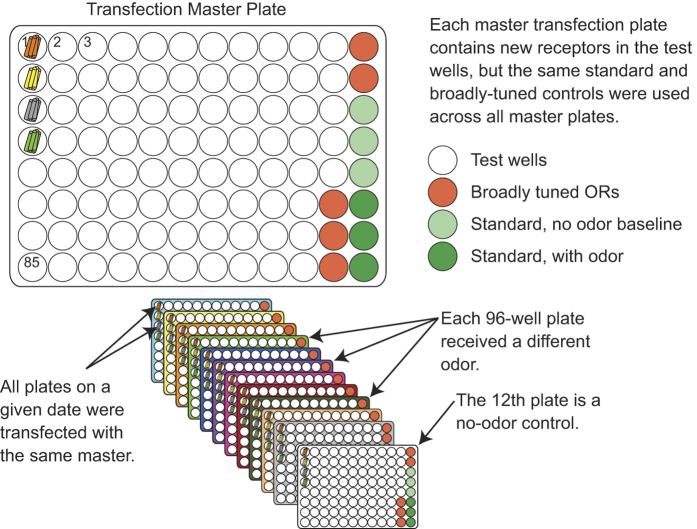
Plate layout for the primary screen. Screens were set up with a master transfection plate for each day. The master transfection plate was used to transfect twelve plates. Each plate was then stimulated with a different odor. Eleven wells were reserved for broadly tuned receptors and a standard to validate the protocol.

***Well***. A number assigned to each well of the 96-well plate. The wells are sequentially numbered with the upper-leftmost well assigned as 1 and the lower-leftmost well assigned as 85 (see Figure 4).

***Concentration***. The concentration of the odorant applied in uM. A ‘9999’ indicates no odor was applied (DMSO was diluted 1:10,000 in CD293).

***Luc***. The number of photons counted by the plate reader when the well was treated with the luciferase substrate. This is the cAMP reporter, and therefore correlates with receptor responses to odorants.

***RL***. The number of photons counted by the plate reader when the well was treated with the Renilla luciferase substrate. This is the constitutively active reporter, which serves as a control for cell death and transfection efficiency.

***OR***. A unique ID for each olfactory receptor clone.

***Odor***. A unique ID for the odorant applied to the well.

***Date***. The date the experiment was run in MM/DD/YY format.

### Data record 2—secondary screen

The raw screening results are presented in a tab-separated values file (Data Citation 1). Each row represents an experiment from a single well.

***Date***. The date the experiment was run in MM/DD/YY format.

***OR***. A unique ID for each olfactory receptor clone.

***Odor***. A unique ID for the odorant applied to the well.

***Concentration***. The concentration of the odorant applied in uM. A ‘0’ indicates no odor was applied (CD293 only). Note that rows for the ‘no odor’ condition will contain an ‘Odor’ label to facilitate pairing controls with the matched experiments at other concentrations.

**NormalizedLuc.** The Luc/RL ratio from each well.

### Data record 3—dose-response

The Luc/RL ratios are presented in a tab-separated values file (Data Citation 1). Each row represents an experiment from a single well. The EC50 for each odor/receptor pair that passed this phase of screening is listed in Table 1 (available online only).

***Concentration***. The molarity applied to the well. Note that the no-odor condition was coded as −12 in this column.

**NormLuc.** The Luc/RL ratio from each well.

***OR***. A unique ID for each olfactory receptor clone.

***Odor***. A unique ID for the odorant applied to the well.

***Date***. The date the experiment was run in MM/DD/YY format.

### Data record 4—receptors

The receptor information is presented in a tab-separated values file (Data Citation 1). Each row represents a single olfactory receptor.

***OR***. A unique ID for the olfactory receptor, used in Data Records 1–3.

***Gene***. The gene name of the olfactory receptor encoded in a given plasmid, followed by the amino acid changes from the hg19 reference sequence for the gene. For example, ‘OR6Y1 V252I’ encodes the gene OR6Y1, but while the hg19 reference sequence has a ‘V’ as the 252nd amino acid, this clone encodes an ‘I’ at position 252. Note that some plasmids were cloned from older builds of the genome, which may start at a different methionine than the current model. These differences from h19 reference may not appear here. Please consult the nucleotide sequence for a more thorough description of differences from the reference sequence.

***NucleotideSeq***. The nucleotide sequence for the olfactory receptor encoded in a given plasmid. Note that the rhodopsin tag is not included in this field.

### Data record 5—odors

The odor information is presented in a tab-separated values file (Data Citation 1). Each row represents a single odor. Further synonyms can be found in a file which correlates all of the CIDs in PubChem with submitted synonyms (ftp://ftp.ncbi.nlm.nih.gov/pubchem/Compound/Extras/CID-Synonym-filtered.gz).

***Odor***. A unique ID for the odorant, used in Data Records 1–3.

***CASRegistryNum***. The Chemical Abstracts Service number for the odorant, when available.

***OdorName***. Common name for the odorant applied to the well.

***CID***. PubChem Compound Identification number, a non-zero integer PubChem accession identifier for a unique chemical structure, when available.

***SMILES***. Simplified Molecular-Input Line-Entry System string, an ASCII string identifier for a unique chemical structure.

## Technical Validation

**Figure 5:**
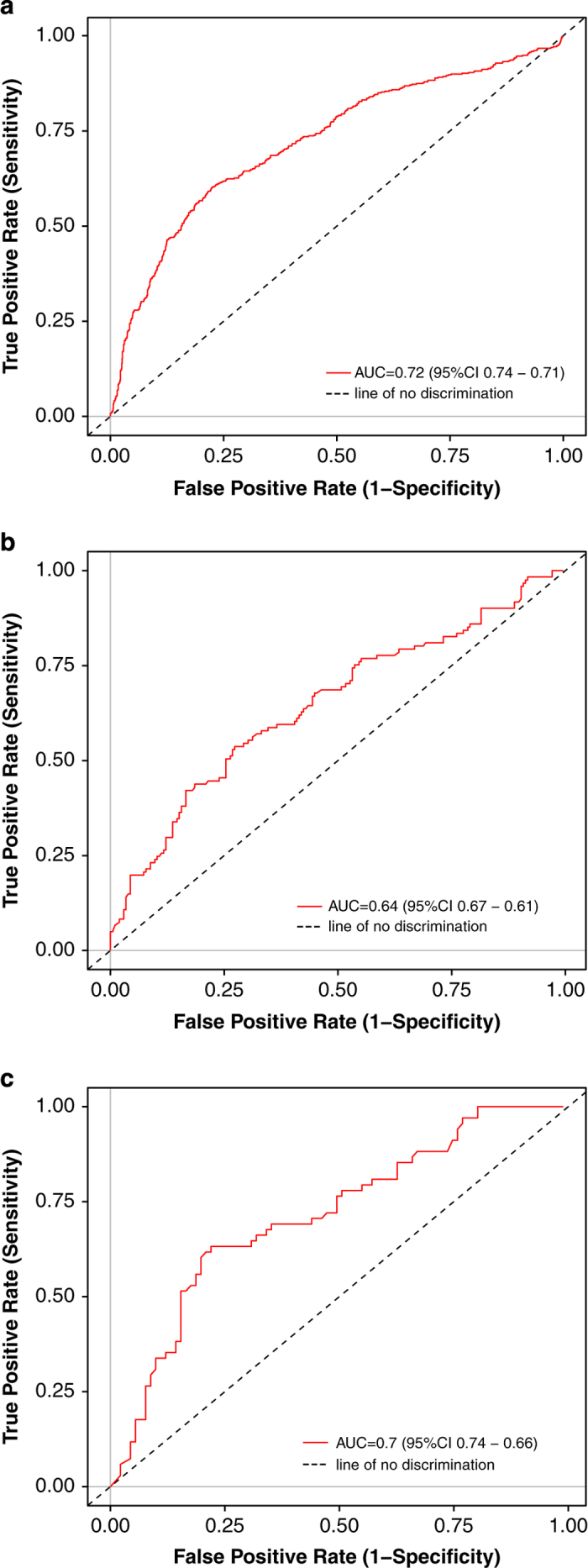
Validation of the screen. An ROC curve indicates that (**a**) the primary screen predicts odor/receptor pairs that pass the secondary screen, (**b**) the primary screen predicts odor/receptor pairs that pass the dose response filter, and (**c**) the secondary screen predicts odor/receptor pairs that pass the dose response filter.

The screen included two types of negative controls. Cells transfected with each receptor clone were challenged with a no-odor stimulation (CD293 alone) to control for baseline receptor activity. Cells transfected with an empty vector were challenged with all of the tested odorants to control for nonspecific activation. All plates in the primary screen included five broadly-tuned odorant receptors and six wells transfected with Olfr544 (also known as MOR42-3 or S6) which served as a standard. Of the six wells transfected with Olfr544, three were challenged with the diluent (CD293), and three were challenged with 10 μM of nonanedioic acid. Rankings from the primary screen consistently predicted results from later screens (Figure 5). The ultimate validation of this assay is prediction of behaviour, and previous results from similar *in vitro* assays have been shown to predict human olfactory perception^12,15,35,36^.

## Usage Notes

We have included R^37^ scripts to facilitate analysis of the data. The included R scripts, Supplementary Files 1 and 2, have been implemented as a hosted Shiny^38^ application (http://www.monell.org/supplemental_files/jmainland/jm0714) to facilitate browsing the data^39–41^. The R markdown file, Supplementary File 3, includes code to carry out routine normalization of the primary screen, fit an ANOVA to data from the secondary screen, fit a sigmoid to the dose-response data, and create Figures 2, 3 and 5 ^42,43^.

## Additional information

Table 1 is only available in the online version of this paper.

**How to cite this article:** Mainland, J. D. *et al.* Human olfactory receptor responses to odorants. *Sci. Data* 2:150002 doi: 10.1038/sdata.2015.2 (2015).

## Supplementary Material



Supplementary Table 1

Supplementary File 1

Supplementary File 2

Supplementary File 3

## Figures and Tables

**Table 1:** Log EC50s for all OR/odor pairs that pass the three statistical criteria outlined in the methods

**OR**	**Odor**	**EC50**	**OdorName**	**Gene**	**Accession**
1030	1341	−5	sandalwood	OR14A16 Indel	KP290123
1034	1101	−4	anisaldehyde	OR6P1	KP290127
1034	1337	−5	phenyl acetaldehyde	OR6P1	KP290127
1037	1379	−3	lyral	OR10J5	KP290130
1037	1379	−5	lyral	OR10J5	KP290130
1042	1285	−3	2-decenal	OR14A2	KP290135
1044	1115	−6	coumarin	OR1C1	KP290137
1044	1328	−6	linalool	OR1C1	KP290137
1062	1286	−5	2-ethylfenchol	OR11A1	KP290155
1067	1300	−5	cis-3-hexen-1-ol	OR2W1	KP290160
1067	1300	−5	cis-3-hexen-1-ol	OR2W1	KP290160
1073	1307	−4	ethyl vanillin	OR2J2 T111A	KP290166
1073	1290	−5	androstenone	OR2J2 T111A	KP290166
1073	1416	−5	butyl anthranilate	OR2J2 T111A	KP290166
1073	1311	−6	eugenol methyl ether	OR2J2 T111A	KP290166
1073	1337	−6	phenyl acetaldehyde	OR2J2 T111A	KP290166
1073	1324	−5	isoeugenol	OR2J2 T111A	KP290166
1073	1300	−5	cis-3-hexen-1-ol	OR2J2 T111A	KP290166
1073	1290	−3	androstenone	OR2J2 T111A	KP290166
1082	1310	−3	eugenol acetate	OR2F1	KP290175
1105	1328	−4	linalool	OR1N2 W23R/V230G/T287M	KP290194
1111	1111	−5	cinnamaldehyde	OR9G1	KP290198
1115	1115	−5	coumarin	OR5P3	KP290202
1119	1287	−6	2-methoxy-4-methylphenol	OR10G4 A9V/M134V/V195E/R235G/K295Q	KP290206
1119	1346	−4	vanillin	OR10G4 A9V/M134V/V195E/R235G/K295Q	KP290206
1119	1346	−3	vanillin	OR10G4 A9V/M134V/V195E/R235G/K295Q	KP290206
1120	1309	−8	eugenol	OR10G7	KP290207
1129	1325	−4	isovaleric acid	OR51E1	KP290215
1129	1325	−3	isovaleric acid	OR51E1	KP290215
1129	1325	−3	isovaleric acid	OR51E1	KP290215
1129	1325	−5	isovaleric acid	OR51E1	KP290215
1134	1326	−4	jasmine	OR4A4	KP290220
1136	1326	−4	jasmine	OR1S2	KP290222
1137	1326	−4	jasmine	OR1S2 I46T	KP290223
1142	1341	−4	sandalwood	OR8D1	KP290227
1142	1396	−5	caramel furanone	OR8D1	KP290227
1155	1325	−3	isovaleric acid	OR4C12 V283L	KP290235
1161	1290	−3	androstenone	OR4F17	KP290239
1183	1191	−7	allyl phenylacetate	OR51L1	KP290258
1193	1326	−5	jasmine	OR4X2	KP290267
1195	1282	−7	(+)-menthol	OR8K3 L122R	KP290269
1195	1281	−6	(−)-menthol	OR8K3 L122R	KP290269
1195	1282	−6	(+)-menthol	OR8K3 L122R	KP290269
1205	1069	−3	propionic acid	OR51E2	KP290277
1206	1295	−4	butyric acid	OR51D1	KP290278
1219	1324	−4	isoeugenol	OR10A6	KP290290
1230	1295	−4	butyric acid	OR5D14	KP290299
1251	1299	−4	cinnamon	OR10AG1	KP290316
1257	1325	−4	isovaleric acid	OR1E1	KP290321
1263	1325	−4	isovaleric acid	OR11H4	KP290326
1265	1316	−4	geranyl acetate	OR4L1 R52S	KP290327
1265	1316	−4	geranyl acetate	OR4L1 R52S	KP290327
1272	1346	−3	vanillin	OR10G3 S73G	KP290332
1272	1346	−3	vanillin	OR10G3 S73G	KP290332
1275	1325	−4	isovaleric acid	OR11H6	KP290335
1277	1326	−3	jasmine	OR4N4	KP290337
1282	1274	−5	1-octanethiol	OR2C1 C149W	KP290341
1282	1203	−6	thioglycolic acid	OR2C1 C149W	KP290341
1282	1337	−5	phenyl acetaldehyde	OR2C1 C149W	KP290341
1285	1310	−5	eugenol acetate	OR4D2 C97S/L187F	KP290344
1288	1190	−3	dihydrojasmone	OR3A1	KP290347
1288	1299	−4	cinnamon	OR3A1	KP290347
1291	1341	−5	sandalwood	OR10H1	KP290349
1298	1290	−5	androstenone	OR7D4	KP290356
1298	1290	−6	androstenone	OR7D4	KP290356
1298	1290	−6	androstenone	OR7D4	KP290356
1298	1290	−5	androstenone	OR7D4	KP290356
1298	1290	−5	androstenone	OR7D4	KP290356
1299	1315	−5	androstadienone	OR7C1	KP290357
1299	1315	−5	androstadienone	OR7C1	KP290357
1299	1290	−4	androstenone	OR7C1	KP290357
1306	1309	−4	eugenol	OR52B6 T36A/L90H/A146T/H149R/V267I	KP290361
1339	1310	−4	eugenol acetate	OR13C3 G2C	KP290384
1350	1328	−4	linalool	OR1N2 W37R/V244G/T301M	KP290391
1351	1295	−4	butyric acid	OR2T1	KP290392
1362	1295	−5	butyric acid	OR5AL1	KP290394
1368	1281	−5	(−)-menthol	OR10X1	KP290399
1376	1316	−4	geranyl acetate	OR1D2	KP290405
1377	1081	−3	geraniol	OR2M7	KP290406
1378	1081	−3	geraniol	OR2M7 F35L/V78A/C178F	KP290407
1387	1316	−6	geranyl acetate	OR2A25	KP290416
1387	1360	−6	quinoline	OR2A25	KP290416
1387	1316	−5	geranyl acetate	OR2A25	KP290416
1402	1307	−4	ethyl vanillin	OR2J2 Y74H/T111A/V146A/T218A	KP290431
1402	1337	−5	phenyl acetaldehyde	OR2J2 Y74H/T111A/V146A/T218A	KP290431
1402	1324	−4	isoeugenol	OR2J2 Y74H/T111A/V146A/T218A	KP290431
1403	1324	−4	isoeugenol	OR2J2	KP290432
1403	1337	−4	phenyl acetaldehyde	OR2J2	KP290432
1403	1307	−4	ethyl vanillin	OR2J2	KP290432
1404	1300	−5	cis-3-hexen-1-ol	OR2J2	KP290433
1404	1311	−7	eugenol methyl ether	OR2J2	KP290433
1404	1324	−5	isoeugenol	OR2J2	KP290433
1404	1337	−6	phenyl acetaldehyde	OR2J2	KP290433
1406	1111	−4	cinnamaldehyde	OR2C1	KP290435
1406	1274	−8	1-octanethiol	OR2C1	KP290435
1407	1274	−6	1-octanethiol	OR2C1	KP290436
1408	1274	−7	1-octanethiol	OR2C1 G16S/C149W/R229H	KP290437
1409	1274	−5	1-octanethiol	OR2C1 P58S/C149W	KP290438
1409	1274	−7	1-octanethiol	OR2C1 P58S/C149W	KP290438
1410	1379	−4	lyral	OR10J5 R233W	KP290439
1411	1416	−6	butyl anthranilate	OR4Q3	KP290440
1411	1309	−6	eugenol	OR4Q3	KP290440
1418	1115	−3	coumarin	OR2B11	KP290447
1418	1192	−3	dicyclohexyl disulfide	OR2B11	KP290447
1418	1342	−5	spearmint	OR2B11	KP290447
1418	1202	−7	coffee difuran	OR2B11	KP290447
1418	1360	−5	quinoline	OR2B11	KP290447
1418	1111	−6	cinnamaldehyde	OR2B11	KP290447
1423	1325	−4	isovaleric acid	OR11H4	KP290452
1432	1326	−4	jasmine	OR2T6 N21D/C23G/S243A	KP290460
1457	1325	−4	isovaleric acid	OR11H6	KP290483
1461	1111	−5	cinnamaldehyde	OR10H2 L40Q	KP290486
1461	1111	−4	cinnamaldehyde	OR10H2 L40Q	KP290486
1462	1324	−4	isoeugenol	OR2S2	KP290487
1462	1324	−4	isoeugenol	OR2S2	KP290487
1463	1299	−5	cinnamon	OR1N1	KP290488
1474	1295	−3	butyric acid	OR2T1 P132L	KP290499
1474	1341	−5	sandalwood	OR2T1 P132L	KP290499
1494	1316	−3	geranyl acetate	OR2M2 R220G/C235R	KP290518
1494	1316	−3	geranyl acetate	OR2M2 R220G/C235R	KP290518
1499	1325	−3	isovaleric acid	OR8U8	KP290523
1502	1278	−6	TMT	OR5K1	KP290526
1502	1278	−9	TMT	OR5K1	KP290526
1502	1278	−6	TMT	OR5K1	KP290526
1502	1278	−9	TMT	OR5K1	KP290526
1502	1278	−9	TMT	OR5K1	KP290526
1502	1311	−8	eugenol methyl ether	OR5K1	KP290526
1502	1278	−9	TMT	OR5K1	KP290526
1505	1328	−4	linalool	OR1N2	KP290529
1522	1324	−5	isoeugenol	OR2AT4	KP290546
1522	1324	−5	isoeugenol	OR2AT4	KP290546
1528	1024	−7	(+)-carvone	OR1A1 R128H	KP290552
1529	1024	−7	(+)-carvone	OR1A1	KP290553
1529	1024	−8	(+)-carvone	OR1A1	KP290553
1530	1024	−7	(+)-carvone	OR1A1 V233M	KP290554
1530	1024	−8	(+)-carvone	OR1A1 V233M	KP290554
1531	1024	−7	(+)-carvone	OR1A1 P285S	KP290555
1531	1024	−8	(+)-carvone	OR1A1 P285S	KP290555
1532	1325	−4	isovaleric acid	OR51E1 S11N	KP290556
1532	1325	−5	isovaleric acid	OR51E1 S11N	KP290556
1532	1325	−5	isovaleric acid	OR51E1 S11N	KP290556
1532	1325	−4	isovaleric acid	OR51E1 S11N	KP290556
1533	1191	−8	allyl phenylacetate	OR51L1 T196I/A207V	KP290557
1534	1191	−8	allyl phenylacetate	OR51L1	KP290558
1535	1191	−7	allyl phenylacetate	OR51L1 I281M	KP290559
1536	1300	−6	cis-3-hexen-1-ol	OR2W1 D296N	KP290560
1571	1111	−5	cinnamaldehyde	OR2C1 G16S/C149W/C169Y/R229H	KP290588
1571	1337	−4	phenyl acetaldehyde	OR2C1 G16S/C149W/C169Y/R229H	KP290588
1571	1274	−4	1-octanethiol	OR2C1 G16S/C149W/C169Y/R229H	KP290588
1572	1300	−5	cis-3-hexen-1-ol	OR2W1 M81V	KP290589
1573	1325	−5	isovaleric acid	OR51E1 K299R	KP290590
1573	1325	−5	isovaleric acid	OR51E1 K299R	KP290590
1574	1290	−5	androstenone	OR7D4	KP290591
1575	1300	−4	cis-3-hexen-1-ol	OR2J1 L12I	KP290592
1581	1388	−5	3-phenyl propyl propionate	OR10A6 V140G/L287P	KP290597
1581	1290	−5	androstenone	OR10A6 V140G/L287P	KP290597
1581	1391	−4	amyl laurate	OR10A6 V140G/L287P	KP290597
1581	1295	−5	butyric acid	OR10A6 V140G/L287P	KP290597
1581	1315	−4	androstadienone	OR10A6 V140G/L287P	KP290597
1581	1388	−8	3-phenyl propyl propionate	OR10A6 V140G/L287P	KP290597
1581	1290	−7	androstenone	OR10A6 V140G/L287P	KP290597
1581	1324	−4	isoeugenol	OR10A6 V140G/L287P	KP290597
1582	1290	−3	androstenone	OR10A6 A117V/V140G/L287P	KP290598
1582	1290	−4	androstenone	OR10A6 A117V/V140G/L287P	KP290598
1585	1310	−4	eugenol acetate	OR10G3	KP290601
1585	1346	−4	vanillin	OR10G3	KP290601
1589	1292	−5	banana	OR10G7	KP290605
1589	1326	−6	jasmine	OR10G7	KP290605
1589	1342	−4	spearmint	OR10G7	KP290605
1589	1309	−6	eugenol	OR10G7	KP290605
1589	1311	−6	eugenol methyl ether	OR10G7	KP290605
1589	1287	−6	2-methoxy-4-methylphenol	OR10G7	KP290605
1589	1332	−6	nutmeg	OR10G7	KP290605
1589	1309	−6	eugenol	OR10G7	KP290605
1589	1317	−6	guaiacol	OR10G7	KP290605
1590	1309	−6	eugenol	OR10G7 T90A	KP290606
1590	1309	−6	eugenol	OR10G7 T90A	KP290606
1591	1309	−6	eugenol	OR10G7 T13M	KP290607
1591	1309	−6	eugenol	OR10G7 T13M	KP290607
1591	1309	−8	eugenol	OR10G7 T13M	KP290607
1593	1111	−6	cinnamaldehyde	OR10H2	KP290609
1593	1316	−3	geranyl acetate	OR10H2	KP290609
1593	1111	−4	cinnamaldehyde	OR10H2	KP290609
1596	1309	−5	eugenol	OR10H5	KP290612
1597	1309	−4	eugenol	OR10H5	KP290613
1603	1299	−5	cinnamon	OR1N1 P18S	KP290618
1604	1316	−8	geranyl acetate	OR2A25	KP290619
1605	1316	−5	geranyl acetate	OR2A25 S75N	KP290620
1606	1316	−8	geranyl acetate	OR2A25 A209P	KP290621
1607	1360	−6	quinoline	OR2A25 S75N/A209P	KP290622
1607	1316	−8	geranyl acetate	OR2A25 S75N/A209P	KP290622
1607	1316	−7	geranyl acetate	OR2A25 S75N/A209P	KP290622
1609	1202	−4	coffee difuran	OR2B11 V198M	KP290624
1609	1360	−5	quinoline	OR2B11 V198M	KP290624
1609	1342	−5	spearmint	OR2B11 V198M	KP290624
1609	1115	−3	coumarin	OR2B11 V198M	KP290624
1611	1360	−5	quinoline	OR2B11 V198M/T293I/D300G	KP290626
1611	1115	−4	coumarin	OR2B11 V198M/T293I/D300G	KP290626
1611	1342	−6	spearmint	OR2B11 V198M/T293I/D300G	KP290626
1611	1202	−4	coffee difuran	OR2B11 V198M/T293I/D300G	KP290626
1612	1111	−4	cinnamaldehyde	OR2B11 I130S/V198M	KP290627
1616	1300	−5	cis-3-hexen-1-ol	OR2J3 I228V/M261I	KP290631
1616	1111	−6	cinnamaldehyde	OR2J3 I228V/M261I	KP290631
1616	1334	−5	octyl aldehyde	OR2J3 I228V/M261I	KP290631
1616	1300	−5	cis-3-hexen-1-ol	OR2J3 I228V/M261I	KP290631
1616	1111	−5	cinnamaldehyde	OR2J3 I228V/M261I	KP290631
1616	1316	−5	geranyl acetate	OR2J3 I228V/M261I	KP290631
1616	1342	−5	spearmint	OR2J3 I228V/M261I	KP290631
1616	1311	−5	eugenol methyl ether	OR2J3 I228V/M261I	KP290631
1616	1300	−6	cis-3-hexen-1-ol	OR2J3 I228V/M261I	KP290631
1617	1300	−4	cis-3-hexen-1-ol	OR2J3 I228V	KP290632
1617	1300	−5	cis-3-hexen-1-ol	OR2J3 I228V	KP290632
1617	1308	−4	ethylene brassylate	OR2J3 I228V	KP290632
1617	1111	−4	cinnamaldehyde	OR2J3 I228V	KP290632
1617	1111	−6	cinnamaldehyde	OR2J3 I228V	KP290632
1617	1316	−5	geranyl acetate	OR2J3 I228V	KP290632
1618	1316	−3	geranyl acetate	OR2J3	KP290633
1619	1316	−4	geranyl acetate	OR2J3 R226Q/I228V/M261I	KP290634
1619	1300	−4	cis-3-hexen-1-ol	OR2J3 R226Q/I228V/M261I	KP290634
1619	1111	−6	cinnamaldehyde	OR2J3 R226Q/I228V/M261I	KP290634
1619	1300	−4	cis-3-hexen-1-ol	OR2J3 R226Q/I228V/M261I	KP290634
1619	1111	−4	cinnamaldehyde	OR2J3 R226Q/I228V/M261I	KP290634
1623	1295	−4	butyric acid	OR2T1	KP290638
1624	1295	−4	butyric acid	OR2T1 I76V	KP290639
1634	1299	−5	cinnamon	OR3A1	KP290642
1635	1307	−4	ethyl vanillin	OR3A1 R125Q	KP290643
1635	1299	−5	cinnamon	OR3A1 R125Q	KP290643
1636	1299	−5	cinnamon	OR3A1	KP290644
1638	1310	−4	eugenol acetate	OR4D2 L29I	KP290646
1638	1310	−4	eugenol acetate	OR4D2 L29I	KP290646
1639	1310	−4	eugenol acetate	OR4D2	KP290647
1639	1310	−4	eugenol acetate	OR4D2	KP290647
1650	1309	−6	eugenol	OR4Q3 F238L	KP290654
1661	1295	−4	butyric acid	OR5AL1 Insertion	KP290661
1666	1295	−4	butyric acid	OR5D14 Q102L/S249A	KP290665
1670	1311	−3	eugenol methyl ether	OR6F1 F215L	KP290668
1673	1295	−4	butyric acid	OR7G2 V263A/F281V	KP290671
1679	1310	−3	eugenol acetate	OR9G1	KP290677
1683	1300	−3	cis-3-hexen-1-ol	OR14J1	KP290679
1686	1316	−4	geranyl acetate	OR1D2	KP290682
1692	1309	−5	eugenol	OR1D5	KP290686
1724	1345	−7	undecanal	OR56A4	KP290707
1727	1024	−8	(+)-carvone	OR8B3 H20R/Q24R/V34I/M114I	KP290709
1727	1339	−6	r-carvone	OR8B3 H20R/Q24R/V34I/M114I	KP290709
1727	1025	−8	(−)-carvone	OR8B3 H20R/Q24R/V34I/M114I	KP290709
1738	1346	−5	vanillin	OR10G4	KP290714
1738	1346	−5	vanillin	OR10G4	KP290714
1739	1346	−4	vanillin	OR10G4	KP290715
1740	1346	−5	vanillin	OR10G4 K295Q	KP290716
1740	1346	−5	vanillin	OR10G4 K295Q	KP290716
1744	1111	−3	cinnamaldehyde	OR2J3 T113A/R226Q/I228V/M261I	KP290717
1744	1111	−6	cinnamaldehyde	OR2J3 T113A/R226Q/I228V/M261I	KP290717
1745	1300	−5	cis-3-hexen-1-ol	OR2J3	KP290718
1747	1078	−6	n-amyl acetate	OR2J3	KP290720
1747	1111	−4	cinnamaldehyde	OR2J3	KP290720
1747	1111	−6	cinnamaldehyde	OR2J3	KP290720
1747	1300	−5	cis-3-hexen-1-ol	OR2J3	KP290720
1761	1290	−5	androstenone	OR7D4 R88W/T133M	KP290723
1761	1290	−4	androstenone	OR7D4 R88W/T133M	KP290723
1761	1290	−4	androstenone	OR7D4 R88W/T133M	KP290723
1761	1290	−3	androstenone	OR7D4 R88W/T133M	KP290723
1762	1290	−3	androstenone	OR7D4 P79L	KP290724
1764	1416	−4	butyl anthranilate	OR2J2	KP290725
1764	1290	−5	androstenone	OR2J2	KP290725
1764	1307	−3	ethyl vanillin	OR2J2	KP290725
1765	1315	−5	androstadienone	OR7C1 V126I/E171K/S210P	KP290726
1768	1315	−5	androstadienone	OR7C1 S99G/V126I/E171K/S210P	KP290729
1781	1290	−4	androstenone	OR10A6 L287P	KP290731
1781	1388	−8	3-phenyl propyl propionate	OR10A6 L287P	KP290731
1784	1300	−5	cis-3-hexen-1-ol	OR2J3	KP290732
